# Pomegranate Juice Alleviates Preeclampsia Symptoms in an L-NAME-Induced Rat Model: A Dose-Dependent Study

**DOI:** 10.3390/nu17071143

**Published:** 2025-03-26

**Authors:** Sasitorn Kerdsuknirund, Atcharaporn Thaeomor, Pakanit Kupittayanant, Panida Khunkaewla, Suthida Chanlun, Rungrudee Srisawat, Pattama Tongdee, Porntip Nimkuntod, Sajeera Kupittayanant

**Affiliations:** 1 School of Preclinical Sciences, Institute of Science, Suranaree University of Technology, Nakhon Ratchasima 30000, Thailand; sasi.kerdsuknirund@gmail.com (S.K.); atcharaporn@sut.ac.th (A.T.); srisawat@sut.ac.th (R.S.); 2 School of Animal Technology and Innovation, Institute of Agricultural Technology, Suranaree University of Technology, Nakhon Ratchasima 30000, Thailand; pakanit@sut.ac.th; 3 Biochemistry-Electrochemistry Research Unit, School of Chemistry, Institute of Science, Suranaree University of Technology, Nakhon Ratchasima 30000, Thailand; kpanida@sut.ac.th; 4 Department of Pathobiology, Faculty of Veterinary Medicine, Khon Kaen University, Khon Kaen 40000, Thailand; sutvir@kku.ac.th; 5 School of Obstetrics and Gynecology, Institute of Medicine, Suranaree University of Technology, Nakhon Ratchasima 30000, Thailand; pattama_t@sut.ac.th; 6 Suranaree University of Technology Hospital, Nakhon Ratchasima 30000, Thailand; porntipnimk@sut.ac.th; 7 School of Internal Medicine, Institute of Medicine, Suranaree University of Technology, Nakhon Ratchasima 30000, Thailand

**Keywords:** preeclampsia, hypertension, proteinuria, placenta, pomegranate juice

## Abstract

**Objective**: This study aimed to evaluate the dose-dependent therapeutic effects of pomegranate juice on preeclampsia symptoms using an L-NAME-induced rat model. **Methods**: Pregnant rats (*n* = 5/group) were assigned to a negative control group or groups receiving L-NAME to induce preeclampsia, with pomegranate juice administered at low, medium, and high doses from gestation day 7 to 20. Maternal parameters, including body weight, systolic blood pressure, urinary protein, and sFlt-1 levels, were monitored. Kidney and placental histology were assessed on gestation day 20. **Results**: L-NAME successfully induced preeclampsia symptoms, including significant maternal weight gain, hypertension, proteinuria, and increased sFlt-1 levels. Pomegranate juice administration alleviated these symptoms in a dose-dependent manner. High doses significantly prevented weight gain from gestation day 14, reduced the systolic blood pressure from gestation day 16, and lowered proteinuria and the sFlt-1 levels by gestation day 18, achieving values comparable to those of normal pregnant controls. Medium doses showed a moderate improvement, particularly in later gestational stages, while low doses had minimal effects. Pomegranate juice also enhanced placental health by increasing the labyrinth depth and reducing endocapillary hypercellularity, contributing to higher fetal and placental birth weights. The dose–response analysis indicated that the kidneys exhibited a stronger response to pomegranate juice than the placenta, suggesting different sensitivity thresholds. **Conclusions**: Pomegranate juice alleviates preeclampsia symptoms in a dose-dependent manner, significantly improving maternal weight regulation, blood pressure, and proteinuria. The therapeutic effects of pomegranate juice are attributed to its high phenolic content, which reduces sFlt-1 and improves placental function. These findings support pomegranate juice as a potential natural intervention for preeclampsia management.

## 1. Introduction

Preeclampsia is a serious pregnancy complication characterized by hypertension, proteinuria, and systemic endothelial dysfunction, posing significant risks to both the mother and fetus. It arises from abnormal placental implantation, leading to an insufficient oxygen supply and the release of anti-angiogenic factors and oxidative stress markers, which drive vascular dysfunction and inflammation. These disruptions contribute to maternal complications such as cerebral thrombosis, pulmonary edema, renal failure, and hepatic dysfunction, as well as fetal complications including intrauterine growth restriction (IUGR), preterm birth, and stillbirth [1]. The development of preeclampsia is largely driven by three interrelated mechanisms: endothelial dysfunction, oxidative stress, and inflammation. Endothelial dysfunction results from an imbalance in angiogenic and anti-angiogenic factors, particularly the overexpression of soluble fms-like tyrosine kinase-1 (sFlt-1), which antagonizes vascular endothelial growth factor (VEGF) and placental growth factor (PlGF), impairing angiogenesis and increasing vascular resistance [2]. Oxidative stress plays a key role, as excessive reactive oxygen species (ROS) impair endothelial function and disrupt nitric oxide (NO) signaling, causing vasoconstriction and reduced placental perfusion. Furthermore, inflammation contributes to preeclampsia progression, as elevated levels of tumor necrosis factor-alpha (TNF-α) and interleukin-6 (IL-6) promote immune dysregulation, exacerbate vascular inflammation, and increase endothelial dysfunction [3].

To study preeclampsia’s pathophysiology and evaluate potential therapies, Nω-nitro-L-arginine methyl ester hydrochloride (L-NAME) is widely used as an experimental model. L-NAME inhibits nitric oxide synthase (NOS), reducing NO production and causing vasoconstriction, hypertension, and proteinuria, effectively mimicking the key characteristics of human preeclampsia [4]. Increased sFlt-1 expression has also been observed in L-NAME-induced preeclampsia models, supporting its use in angiogenic imbalance research [5,6].

The current treatments for preeclampsia remain limited, prompting research into natural bioactive compounds with antioxidant, anti-inflammatory, and antihypertensive properties. Several plant-derived compounds have shown promise in mitigating preeclampsia symptoms. Quercetin supplementation has been found to reduce hypertension in L-NAME-induced preeclampsia [7], curcumin prevents systolic blood pressure elevation in preeclamptic rats [8], and resveratrol improves placental function and reduces proteinuria in preeclampsia models [9]. Artemisinin supplementation has been shown to lower blood pressure, reduce proteinuria, and improve the placental morphology [10], while plant flavonoids have been reported to restore the angiogenic balance, enhance renal function, and alleviate oxidative stress [11,12]. Given the major role of oxidative stress in preeclampsia’s pathogenesis, natural compounds with strong antioxidant properties could be key in alleviating disease symptoms by restoring vascular function and angiogenic homeostasis [13].

Among these natural interventions, pomegranate (*Punica granatum* L.) juice has gained increasing attention due to its high content of antioxidants, polyphenols, and flavonoids, which have been extensively studied for their anti-inflammatory, antihypertensive, and cardioprotective effects [14]. Pomegranate juice is particularly rich in phenolic compounds, which contribute to cardiovascular health by reducing oxidative stress and inflammation. Clinical studies have demonstrated that long-term pomegranate juice consumption lowers blood pressure and reduces lipid peroxidation in patients with arterial stenosis, and its cardioprotective effects have been validated in individuals with ischemic heart disease and those undergoing dialysis [15,16,17].

Pomegranate juice contains several bioactive polyphenols, including punicalagin, ellagic acid, and anthocyanins, which contribute to its therapeutic effects in preeclampsia. Punicalagin is the most abundant polyphenol in pomegranate juice and has been studied for its antioxidant, anti-inflammatory, and antihypertensive properties. It has been shown to reduce oxidative stress, improve endothelial function, and restore angiogenic balance—key factors in preeclampsia’s pathophysiology. Studies on L-NAME-induced preeclampsia models indicate that punicalagin supplementation significantly reduces the systolic, diastolic, and mean arterial blood pressure; restores the angiogenic balance by upregulating VEGF and downregulating sFlt-1; and enhances the placental NO levels, promoting vascular function [18]. Additionally, punicalagin decreases oxidative stress markers, improves the placental antioxidant capacity, and restores catalase activity, which is elevated in preeclampsia, further supporting its protective role in endothelial dysfunction [18]. Ellagic acid, another major bioactive polyphenol in pomegranate juice, has demonstrated antioxidant, anti-inflammatory, and anti-apoptotic effects. It plays a role in reducing oxidative stress and inflammation in endothelial cells, which may contribute to an improvement in placental function and fetal outcomes in preeclampsia models. Ellagic acid has been shown to enhance NO’s bioavailability, which could counteract L-NAME-induced vasoconstriction, thereby improving vascular function in preeclampsia [19]. Anthocyanins, a subclass of flavonoids found in pomegranate juice, exhibit strong antioxidant and vasoprotective properties. Studies suggest that anthocyanins contribute to endothelial function by reducing vascular inflammation and promoting vasodilation. In preeclampsia models, anthocyanins have been reported to enhance the placental antioxidant defenses, decrease oxidative stress, and improve fetal development [20].

Given its rich polyphenol content and multiple protective mechanisms, this study hypothesizes that pomegranate juice can alleviate preeclampsia symptoms by improving maternal and fetal outcomes. Specifically, this study aims to investigate the effects of pomegranate juice supplementation in an L-NAME-induced preeclampsia rat model, focusing on its impacts on maternal hypertension, proteinuria, weight gain, the placental and renal morphologies, and fetal outcomes. By elucidating how pomegranate juice modulates these parameters and examining its dose-dependent effects, this research seeks to provide novel insights into natural therapeutic strategies for the management of preeclampsia.

## 2. Materials and Methods

### 2.1. Ethics Statement

This study involving animal subjects was conducted in compliance with the 3R principles (Replacement, Reduction, and Refinement) to ensure ethical and humane animal use. The experimental design was structured to minimize the number of animals used while maintaining statistical rigor, refine the procedures to reduce animal distress, and avoid unnecessary animal use by utilizing validated preclinical models. Every effort was made to optimize the housing conditions and monitoring and use humane endpoints to enhance animal welfare.

All procedures were conducted in accordance with the Animals for Scientific Purposes Act 2015, Thailand [21] and approved by the Institutional Animal Care and Use Committee (IACUC) of Suranaree University of Technology (SUT) under protocol number SUT-IAUCUC-0012/2022. The care and handling of the animals followed the Guide for the Care and Use of Laboratory Animals (National Research Council) to ensure ethical and responsible animal research practices.

### 2.2. Pomegranate Juice Preparation and Total Phenolic Measurement

Pomegranates were sourced from a pomegranate farm in Pak Chong District, Nakhon Ratchasima Province, Thailand. The variety was authenticated by the Bangkok Forest Herbarium (BKF), Thailand (BKF No. 188578). The pomegranates were washed and peeled, and the seeds were juiced using a pomegranate juicer. The pomegranate juice used in this study was a kind gift. The juice was filtered through eight layers of cloth and stored in 500 mL bags at −20 °C. Before use, the frozen juice was thawed under running water and centrifuged at 4000 RCF at 4 °C for 15 min. The °Brix level was measured. The juice was concentrated using microwave evaporation at 450 W, aiming for a concentration of 60.5 °Brix. The yield percentage was calculated, and the concentrated juice was stored in light-protected bottles at 4 °C. The total phenolic content was measured using the Folin–Ciocalteu method. The absorption spectra of the pomegranate juice were compared with those of gallic acid as a reference standard compound. The total phenolic content of the concentrated pomegranate juice obtained in this study was 5223.97 mg/L, which is higher than that reported in a previous study [15] that suggested that high levels of phenolic compounds are a primary source of natural antioxidants. The adjusted doses (low, medium, and high) of pomegranate juice in this study were modified and calculated based on a previous study in humans [22].

### 2.3. Animal Preparation

Female rats weighing 200–250 g, aged 3–6 months, were used in this study, totaling 65 rats, taking into account the exclusion criteria, such as sterility, illness, or death during the experiment. The rats were housed at the SUT Animal Facility under controlled conditions: temperature 20–25 °C, relative humidity 30–70%, and a 12/12 h light–dark cycle. They were acclimatized to the environment for one week before the experiment began.

### 2.4. Calculation of Pomegranate Juice Dosage for Experimental Animals

The animal dosage for pomegranate juice in this study was determined based on multiple key considerations to ensure both physiological relevance and safety. First, we utilized the Human Equivalent Dose (HED) conversion method, incorporating total phenolic content data from the previous literature [23], to align the administered doses with human consumption levels. This approach ensures that the dosage selected for the rodent model is translationally meaningful. Second, safety margins were incorporated following the Organisation for Economic Cooperation and Development (OECD) guidelines for toxicity studies [24], ensuring that the doses remained within a safe range for pregnant animals. Finally, we reviewed empirical data from previous studies on the therapeutic effects of pomegranate juice and other natural antioxidants in preeclampsia models to establish dose ranges that were expected to elicit observable therapeutic effects while minimizing the potential toxicity. A simplified summary of the key dosage determination steps is presented in Table 1, while detailed calculations are provided in the Supplementary Materials.

In Table 2, the adjusted dosages of pomegranate juice for the experimental animals, including the corresponding HED and the volumes of concentrated pomegranate juice and clean water used, are shown.

### 2.5. Experimental Design

Female rats in estrous (confirmed by vaginal smear cytology) were mated with male rats in a 2:1 ratio. Pregnancy was confirmed by the presence of vaginal plugs and sperm cells in the vaginal smear, marking day 0 of pregnancy. Mating sessions were synchronized with the optimal time for plug detection (9:00–12:00 a.m.). Ten male rats were used for mating, housed individually. Female rats were checked daily for plugs, and, once found, they were separated and housed individually.

The female rats were randomly divided into five groups, each containing 10–15 rats [13].

1. Negative control group (9 rats): Received a subcutaneous injection of saline solution (0.5 mL/100 g body weight) daily from gestation day 7 to day 20, along with the oral administration of distilled water (1 mL/rat/day).
2. L-NAME group (preeclamptic control group) (14 rats): Received a subcutaneous injection of the nitric oxide synthase inhibitor L-NAME (10 mg/0.5 mL saline/100 g body weight [27]) daily from gestation day 7 to day 20 to induce preeclampsia, along with the oral administration of distilled water (1 mL/rat/day).
3. Low-dose pomegranate juice group (13 rats): Received L-NAME (same dosage as group 2) along with the oral administration of low-dose pomegranate juice (3.1 mL/kg body weight/day).
4. Medium-dose pomegranate juice group (13 rats): Received L-NAME (same dosage as group 2) along with the oral administration of medium-dose pomegranate juice (4.7 mL/kg body weight/day).
5. High-dose pomegranate juice group (10 rats): Received L-NAME (same dosage as group 2) along with the oral administration of high-dose pomegranate juice (7.2 mL/kg body weight/day).

All treatments were administered daily from day 7 to day 20 of pregnancy.

To minimize pain, suffering, and distress in the experimental animals, several interventions were implemented throughout the study. All procedures were conducted in accordance with institutional guidelines for animal care and use and were approved by the relevant ethics committee. The handling of the animals was performed gently to reduce stress, and subcutaneous injections were administered using fine-gauge needles to minimize discomfort. Oral administrations were conducted carefully to avoid aspiration or injury.

The animals were monitored twice daily for signs of distress, including changes in behavior (e.g., decreased activity, abnormal posture), physical appearance (e.g., piloerection, unkempt fur), and physiological indicators (e.g., labored breathing, weight loss). Any signs of pain or discomfort were documented, and animals exhibiting severe distress were provided with appropriate interventions or, if necessary, humanely euthanized.

Humane endpoints were established for the study to prevent unnecessary suffering. Criteria for humane endpoints included a loss of more than 15% in body weight, inability to eat or drink, severe respiratory distress, unresponsiveness to stimuli, or any signs of severe pain that could not be alleviated. Animals meeting these criteria were euthanized using an overdose of anesthetic agents, followed by a secondary method to confirm death.

Expected adverse events included mild discomfort from injections and potential side effects of L-NAME administration, such as hypertension and proteinuria, consistent with the preeclampsia model. Unexpected adverse events, if any, were recorded and reviewed by the research team to determine the appropriate course of action.

### 2.6. Measurement Parameters for Pomegranate Juice Effects

#### 2.6.1. Blood Pressure Measurement

The systolic blood pressure was measured using a tail cuff sphygmomanometer on days 6, 10, 14, 16, 18, and 20 of pregnancy, between 8:00 a.m. and 10:00 a.m. Rats’ tails were warmed to 38 °C locally under a heat lamp to acclimate before measurements. For each rat, the systolic blood pressure was measured 12 times, and the three most consistent values (variability < 6 mmHg) were averaged.

#### 2.6.2. Urine Protein Measurement

Urine samples were collected on days 6, 10, 14, 16, and 18 of pregnancy using metabolic cages (Tecniplast™, TECNIPLAST S.p.A., Varese, Italy). An automated dipstick device (UriDoctorTM VET, ZUELLIG PHARMA Ltd., Bangkok, Thailand) was used for urine protein analysis, with values graded on a scale from 0 to 4 (0 = negative/trace, 1 = 30 mg/dL, 2 = 100 mg/dL, 3 = 300 mg/dL, 4 = 1000 mg/dL) [28,29].

#### 2.6.3. sFLT-1 Levels

All rats were euthanized on day 20 of pregnancy using CO_2_ asphyxia, and blood was collected from a cardiac puncture. Serum samples were obtained by centrifuging the blood at 1500 r/min for 10 min at 4 °C and were assayed for the sFLT-1 level using a commercial sandwich ELISA kit (FineTest^®^, Wuhan Fine Biotech Co., Ltd., Hubei, China), following the manufacturer’s instructions.

#### 2.6.4. Weighing of Newborns and Placentas and Calculation of Placental Index

The newborns were weighed shortly after delivery using electronic scales. Similarly, the placentas were weighed soon after delivery, with the membranes and umbilical cord still attached. Blood and clots were removed using an absorbent cotton pad before weighing. The placental index was determined by dividing the placental weight by the birth weight, with both measurements recorded in grams.

#### 2.6.5. Placental and Kidney Histology

Maternal placenta and kidney samples were collected immediately after delivery. The tissues were carefully excised and immediately placed in 10% neutral buffered formalin to ensure proper fixation. The samples were then processed using standard histological techniques, including dehydration through a graded series of alcohols, clearing in xylene, and embedding in paraffin wax. Sections were cut to a 4–5 µm thickness and stained with hematoxylin and eosin (H&E) for microscopic examination. The 3DHISTECH Pannoramic MIDI II slide scanner (3DHISTECH Ltd., Budapest, Hungary), equipped with the Pannoramic Scanner software version 3.0 and Slide Viewer version 2.7 for image capture and area measurement (3DHISTECH Ltd., Budapest, Hungary), was used for histological analysis. For each section, three randomly selected fields of view were photographed for histological assessment. An experienced pathologist performed a blind assessment of the placenta and renal histology. Histological alterations in the placenta were quantified by measuring the placenta diameter of the labyrinthine area and junctional zone in micrometers (µm) and then calculating the ratio of the labyrinthine to the junctional zone [30]. Kidney endocapillary hypercellularity was assessed and graded on a scale from 0 to 4 (0 = absent, 1 ≤ 25% present, 2 = 25–50% present, 3 = 50–75% present, 4 ≥ 75% present) [31].

### 2.7. Data Analysis

The data are presented as means ± standard deviations (SD), and ‘*n*’ represents the number of animals. All data were published using the Microcal Origin software version 8.5 (©OriginLab Co., Northampton, MA, USA). One-way analysis of variance (ANOVA) followed by Tukey’s post hoc test was utilized to compare the mean values among different treatment groups, using SPSS version 17.0 from SPSS Inc., Chicago, IL, USA. A probability level of less than 5% (*p* < 0.05) was considered statistically significant.

## 3. Results

### 3.1. L-NAME Successfully Induced Preeclampsia

**Figure 1:**
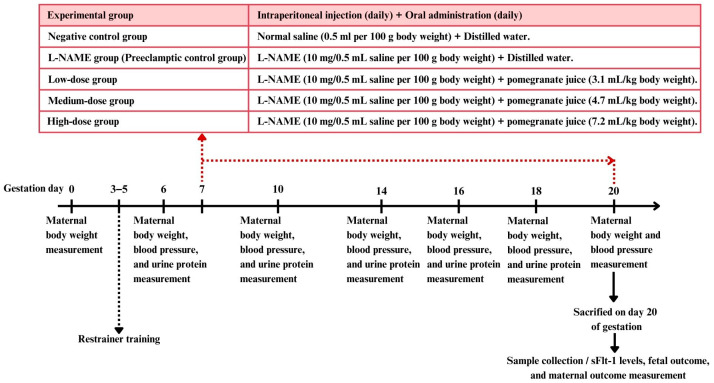
Experimental design and timeline of measurements. The figure illustrates the experimental setup, including the grouping of pregnant rats and the schedule of treatments and measurements.

Figure 1 illustrates the experimental setup, treatment groups, and measurement timeline.

**Figure 2:**
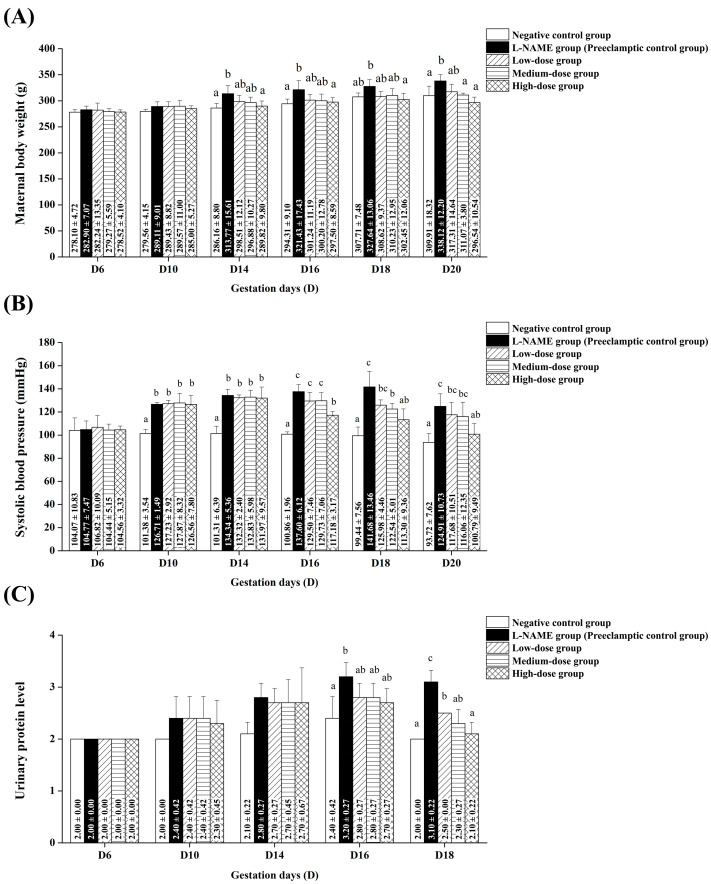
L-NAME-induced preeclampsia-like features and the effects of pomegranate juice on maternal health parameters. (**A**) Maternal body weight across gestation days 6, 10, 14, 18, and 20. Comparison between the control group, L-NAME group (preeclamptic control), and pomegranate juice-treated groups (low, medium, and high doses). (**B**) Systolic blood pressure measurements across gestation days 6, 10, 14, 18, and 20, showing the progression of hypertension in preeclamptic rats and the effects of pomegranate juice in reducing blood pressure. (**C**) Urinary protein concentration as an indicator of proteinuria across gestation days 6, 10, 14, and 18. Elevated levels in the L-NAME group indicate preeclampsia, while pomegranate juice administration reduces proteinuria in a dose-dependent manner. Data are presented as mean ± standard deviation (SD) and were analyzed by one-way ANOVA followed by Tukey’s post hoc test (*p* < 0.05; *n* = 5 per group). Groups bearing different superscript letters on the bars exhibited significant differences (*p* < 0.05).

The preeclampsia model was successfully established, as evidenced by maternal weight gain (percentage of original body weight), hypertension (systolic blood pressure ≥ 140 mmHg), and proteinuria (urinary protein concentration > 300 mg/mL) following L-NAME injections [32,33] (Figure 2).

### 3.2. Pomegranate Juice Alleviated Preeclampsia

#### 3.2.1. Effects of Pomegranate Juice on Maternal Weight Gain

Figure 2A illustrates the effects of pomegranate juice on maternal weight gain in preeclamptic rats. The preeclamptic control group (L-NAME-treated rats without pomegranate juice) showed a gradual increase in body weight, with a significant difference from the normal pregnant control group from gestation day 14 onward (*p* < 0.05). The low-dose group showed no significant difference in body weight compared to either the normal pregnant control group or the preeclamptic control group (*p* > 0.05) during gestation days 14–20. The medium-dose group showed no significant difference in body weight on gestation days 14–18; however, on gestation day 20, the body weight was significantly lower than in the preeclamptic control group (*p* < 0.05) and not significantly different from that of the normal pregnant control group (*p* > 0.05). The high-dose group experienced no excessive weight gain from gestation day 14 onward, with the body weight being significantly lower than that of the preeclamptic control group (*p* < 0.05) but not significantly different from that of the normal pregnant control group (*p* > 0.05).

#### 3.2.2. Effects of Pomegranate Juice on Systolic Blood Pressure

Figure 2B shows the effects of pomegranate juice on the systolic blood pressure. The preeclamptic control group exhibited a significant increase in systolic blood pressure compared to the normal pregnant control group from gestation day 10 onward (*p* < 0.05). The low-dose group showed no significant difference in systolic blood pressure compared to either the normal pregnant control group or the preeclamptic control group (*p* > 0.05) on gestation days 10–20. The medium-dose group exhibited a significant reduction in systolic blood pressure, with values still higher than the normal pregnant control group (*p* < 0.05) on gestation days 14–16; however, by gestation days 18 and 20, the difference was no longer significant (*p* > 0.05). The high-dose group exhibited no increase in systolic blood pressure from gestation day 16 onward, with values significantly lower than in the preeclamptic control group (*p* < 0.05) and not significantly different from those of the normal pregnant control group (*p* > 0.05) on gestation days 18 and 20.

#### 3.2.3. Effects of Pomegranate Juice on Urinary Protein Levels

Figure 2C illustrates the effects of pomegranate juice on the urinary protein levels. The preeclamptic control group exhibited a significant increase in urinary protein compared to the normal pregnant control group from gestation day 16 onward (*p* < 0.05). No significant difference was observed between the pomegranate-treated groups and the preeclamptic or normal pregnant control groups (*p* > 0.05) during gestation days 10–16. However, on gestation day 18, the urinary protein levels in all pomegranate-treated groups were significantly lower than in the preeclamptic control group (*p* < 0.05). Additionally, in the medium-dose and high-dose groups, the urinary protein levels did not significantly differ from those in the normal pregnant control group (*p* > 0.05), suggesting a dose-dependent protective effect.

#### 3.2.4. Effects of Pomegranate Juice on sFlt-1 Levels

**Figure 3:**
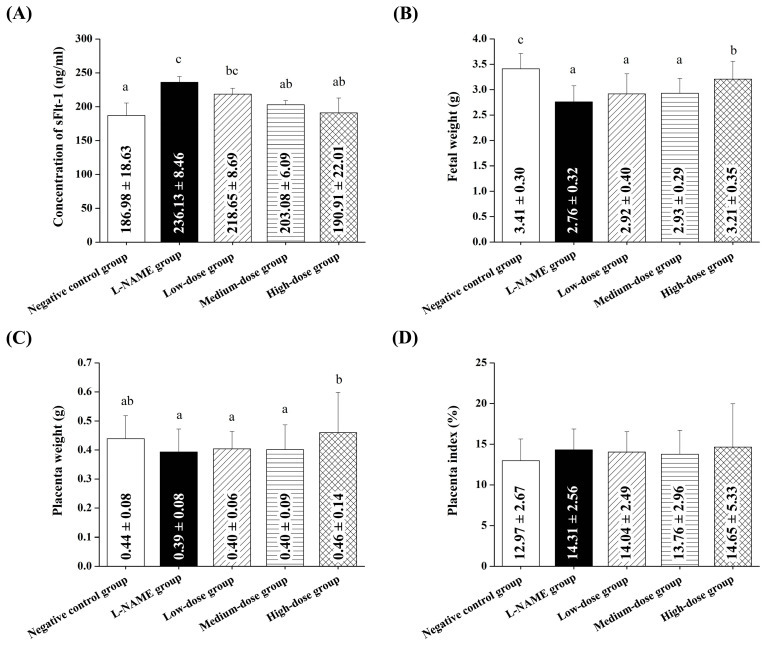
Effects of pomegranate juice on sFlt-1 levels, fetal weight, placental weight, and placental index in pregnant rats with L-NAME-induced preeclampsia. (**A**) sFlt-1 concentration (ng/mL) in maternal serum on gestation day 20. L-NAME significantly increased the sFlt-1 levels compared to the control group, while pomegranate juice administration reduced the sFlt-1 levels in a dose-dependent manner. (**B**) Fetal weight (g) on gestation day 20. L-NAME-treated rats exhibited significantly lower fetal weights compared to controls. High-dose pomegranate juice significantly improved the fetal weight. (**C**) Placental weight (g) on gestation day 20. Preeclamptic rats showed a reduced placental weight, which was partially restored by high-dose pomegranate juice treatment. (**D**) Placental index (%) on gestation day 20. No significant differences were observed among the groups, indicating that the placental weight changes were proportional to the fetal weight changes. Data are presented as the mean ± standard deviation (SD) and were analyzed using one-way ANOVA, followed by Tukey’s post hoc test (*p* < 0.05). Different superscript letters indicate statistical significance (*p* < 0.05) between the groups.

Figure 3A shows the effects of pomegranate juice on the sFlt-1 levels, a key biomarker of preeclampsia. The L-NAME group exhibited significantly elevated sFlt-1 levels compared to the control group (*p* < 0.05), confirming successful preeclampsia induction. The low-dose pomegranate juice group exhibited a non-significant reduction in sFlt-1 levels compared to the L-NAME group (*p* > 0.05). The medium-dose group demonstrated a significant reduction in sFlt-1 levels, particularly at the later treatment stages (*p* < 0.05). The high-dose pomegranate juice group exhibited the most substantial effect, reducing the sFlt-1 concentrations to levels comparable to those of the normal control group (*p* > 0.05). These findings suggest the dose-dependent therapeutic potential of pomegranate juice in mitigating elevated sFlt-1 levels induced by L-NAME.

#### 3.2.5. Effects of Pomegranate Juice on Fetal and Placental Parameters

On gestation day 20, the L-NAME group exhibited significantly lower fetal and placental birth weights than the control group (*p* < 0.05) (Figure 3B,C). The high-dose pomegranate juice group exhibited a significantly improved fetal birth weight and placental weight (*p* < 0.05), while the placenta index showed no significant difference among the groups (*p* > 0.05, Figure 3D).

#### 3.2.6. Effects of Pomegranate Juice on Placental Morphology

**Figure 4:**
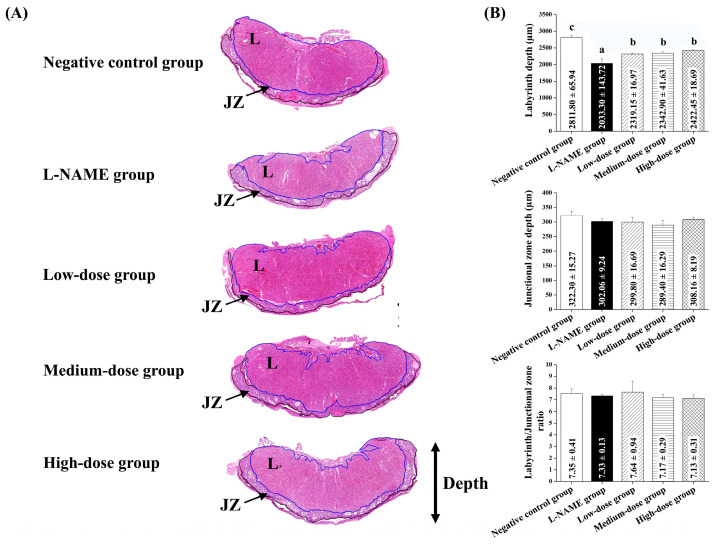
Histopathological analysis of placentas from pregnant rats with L-NAME-induced preeclampsia, with or without pomegranate juice treatment. (**A**) Representative histological images of placentas stained with hematoxylin and eosin (H&E), showing the placental labyrinth (L) and junctional zone (JZ). (**B**) Quantification of placental parameters, including labyrinth depth (top graph), junctional zone depth (middle graph), and labyrinth-to-junctional zone ratio (bottom graph). L-NAME significantly reduced the labyrinth depth compared to the control group (*p* < 0.05), while pomegranate juice treatment improved the labyrinth depth in a dose-dependent manner (*p* < 0.05). No significant differences were observed in the junctional zone depth or the labyrinth-to-junctional zone ratio among the groups (*p* > 0.05). Data are presented as mean ± standard deviation (SD) and were analyzed using one-way ANOVA, followed by Tukey’s post hoc test (*p* < 0.05). Different superscript letters indicate statistical significance (*p* < 0.05) between groups.

Figure 4A illustrates the placental labyrinth depth. The placental labyrinth depth was significantly reduced in the L-NAME group compared to the control group (*p* < 0.05, Figure 4B, top graph). All pomegranate-treated groups exhibited a significant improvement in the placental labyrinth depth (*p* < 0.05, Figure 4B, top graph). However, there were no significant differences in the placental junctional zone depth (middle graph) or the labyrinth-to-junctional zone ratio (bottom graph) among the groups (*p* > 0.05, Figure 4B).

#### 3.2.7. Effects of Pomegranate Juice on Renal Morphology

**Figure 5:**
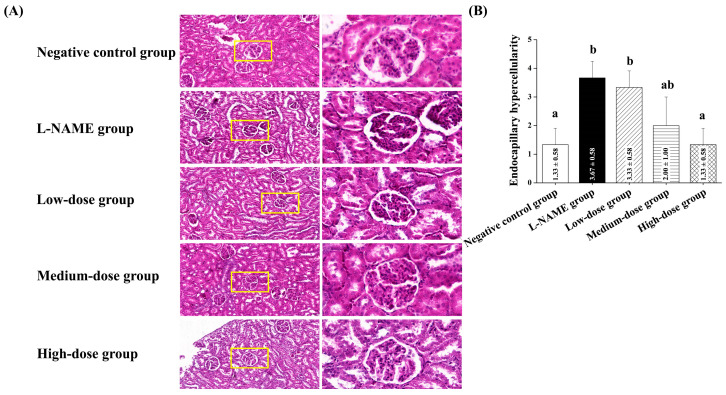
Histopathological analysis of renal tissue in pregnant rats with L-NAME-induced preeclampsia, with or without pomegranate juice treatment. (**A**) Representative kidney sections (left, scale bar: 200 μm; right, scale bar: 50 μm) stained with H&E, demonstrating endocapillary hypercellularity, a marker of renal damage in preeclampsia. The area highlighted by the yellow frame in the left image is shown at higher magnification on the right. (**B**) L-NAME-treated rats exhibited significantly increased endocapillary hypercellularity compared to the control group (*p* < 0.05). Pomegranate juice administration, particularly at high doses, significantly reduced endocapillary hypercellularity compared to the L-NAME group (*p* < 0.05). Data are presented as mean ± standard deviation (SD) and were analyzed using one-way ANOVA, followed by Tukey’s post hoc test (*p* < 0.05). Different superscript letters indicate statistical significance (*p* < 0.05) between groups.

Figure 5A presents histopathological images of the renal tissue. Endocapillary hypercellularity was significantly increased in the L-NAME group compared to the control group (*p* < 0.05, Figure 5B), indicating renal damage. Interestingly, high-dose pomegranate juice significantly reduced endocapillary hypercellularity compared to the preeclamptic control group (*p* < 0.05, Figure 5B), suggesting improved renal vascular function.

#### 3.2.8. Dose-Dependent Response

**Figure 6:**
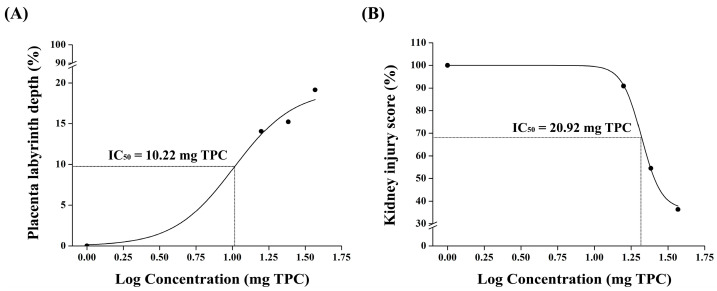
Dose–response analysis of pomegranate juice’s effects on placental labyrinth depth and kidney injury scores in L-NAME-induced preeclampsia. (**A**) The left panel illustrates the dose–response curve for the placental labyrinth depth, showing a sigmoidal increase with an IC_50_ of 10.22 mg TPC/kg, indicating that a lower pomegranate juice dose is sufficient to achieve 50% of the maximal improvement in the placental structure. (**B**) The right panel depicts the dose–response curve for renal injury (endocapillary hypercellularity score), demonstrating a sigmoidal decline with an IC_50_ of 20.92 mg TPC/kg, suggesting that a higher pomegranate juice dose is required to achieve a 50% reduction in renal damage. Data points were derived from experimental groups receiving different pomegranate juice doses (low, medium, and high) and fitted using a non-linear regression model. The difference in IC_50_ values between the placental and renal responses highlights the organ-specific sensitivity to pomegranate juice treatment, with the placenta exhibiting a more pronounced response at lower doses, whereas the kidney requires a higher concentration for significant histological improvement.

Figure 6 illustrates the dose–response analysis, which provides critical insight into the efficacy of pomegranate juice in improving the placental and kidney morphologies in the L-NAME-induced preeclampsia model. The dose–response curve for the placental labyrinth depth demonstrates a sigmoidal increase in response to pomegranate juice administration, with an IC_50_ value of 10.22 mg TPC/kg, indicating that a relatively low dose of pomegranate juice is sufficient to achieve 50% of the maximal improvement in the placental structure (Figure 6A). The curve trajectory indicates a dose-dependent improvement, with higher doses further enhancing the labyrinth depth, although the observed plateau effect at higher doses suggests the possible saturation of the biological response, where no additional benefits are observed beyond a certain concentration.

In contrast, the kidney score dose–response curve follows a sigmoidal decline, demonstrating that pomegranate juice reduces renal damage in a dose-dependent manner, with an IC_50_ value of 20.92 mg TPC/kg, which is higher than that observed for placental improvement (Figure 6B). This suggests that a higher pomegranate juice concentration is required to achieve a 50% reduction in renal hypercellularity compared to placental recovery, implying that kidney injury in preeclampsia may be less responsive or require greater antioxidant intervention to restore normal histological features. The steep decline in hypercellularity at increasing doses indicates that pomegranate juice exerts protective effects on the renal structure.

## 4. Discussion

Our study demonstrates that pomegranate juice alleviates preeclampsia symptoms in a dose-dependent manner, with significant effects on maternal weight gain, hypertension, proteinuria, and sFlt-1 levels in pregnant rats induced with L-NAME. The timing of these effects and the dose–response relationship suggest its therapeutic potential in mitigating the adverse consequences of preeclampsia. Notably, the high-dose group exhibited the most pronounced protective effects, significantly reducing maternal weight gain and hypertension from mid-gestation onward. These findings position pomegranate juice as a promising natural intervention to alleviate maternal complications associated with preeclampsia.

The observed improvements align with the known pathophysiology of L-NAME-induced preeclampsia, where oxidative stress disrupts placental function, leading to systemic complications. The time-dependent response to pomegranate juice suggests that its antioxidative properties help to counteract oxidative stress-induced damage, supporting its role as an early intervention strategy [31]. The improvements in the placental morphology and fetal outcomes suggest that pomegranate juice enhances placental function, potentially by modulating oxidative stress and endothelial dysfunction. The observed increase in the placental labyrinth depth and reduction in endocapillary hypercellularity further indicate a protective role against L-NAME-induced placental damage. These findings align with previous research highlighting the impact of polyphenol-rich interventions on vascular health and angiogenesis in pregnancy-related disorders. Therefore, pomegranate juice alleviates preeclampsia symptoms in a dose-dependent manner, with high doses showing the most significant effects. The therapeutic action of pomegranate juice begins approximately one week after the induction of preeclampsia, highlighting its potential for early intervention. By counteracting oxidative stress, pomegranate juice improves placental function and reduces the maternal and fetal complications associated with preeclampsia.

Hypertension and proteinuria, which are common features of preeclampsia, exacerbate fluid retention and edema, resulting in significant weight gain [3]. In our study, there was no significant increase in maternal weight in any of the groups administered pomegranate juice at low, medium, and high doses compared to the L-NAME group. This suggests that pomegranate juice can prevent the weight gain typically associated with preeclampsia. The mechanism behind this effect may be related to pomegranate juice’s ability to prevent fluid retention, hypertension, and proteinuria, as discussed in detail below.

The most notable finding of our study was the effect of pomegranate juice on the systolic blood pressure. All pomegranate juice-treated groups demonstrated no increase in systolic blood pressure, with the medium-dose pomegranate juice group exhibiting a particularly significant preventive effect against blood pressure elevation. This suggests a dose-dependent response to pomegranate juice, with the medium dose being the most effective in managing hypertension in this L-NAME-induced preeclampsia model. Ożarowski et al. found that several studies on preeclampsia have reported similar findings with high-total-phenolic compounds [13] such as Euterpe oleracea (1808.00–3437.00 mg GAE/100 g extract) [34], *Uncaria rhynchophylla* (241.9 ± 17.5 mg GAE/g extract) [35], *Vitis vinifera* (82.8 mg GAE/g extract) [36], *Uncaria rhynchophylla* (241.9 ± 17.5 mg GAE/g extract) [36], and *Vitis vinifera* (82.8 mg GAE/g extract) [37]. These studies support the notion that plant phenolic compounds can be used to effectively manage hypertension associated with preeclampsia. Our study aligns with these findings, further suggesting that natural compounds can be beneficial in managing hypertension associated with preeclampsia.

The urinary protein levels demonstrated a decreasing trend with low and medium doses of pomegranate juice treatment, while the high-dose pomegranate juice notably reduced the urinary protein levels to match those of the negative control group. This suggests the potential for pomegranate juice to reduce proteinuria, consistent with findings from other studies. For instance, Smyatskaya et al. reported that grapes and wine products containing high levels of phenolic compounds such as resveratrol significantly reduced proteinuria in L-NAME-induced preeclampsia rats [38], highlighting the potential of natural compounds to modulate renal function and protein excretion in preeclampsia models [10]. Our results provide promising insights into the anti-hypertensive effects of pomegranate juice and its potential role in reducing proteinuria.

Our findings further confirmed that pomegranate juice administration produced beneficial effects at the organ level, particularly in the placenta and kidneys, which are critical in managing preeclampsia symptoms.

Pomegranate juice administration resulted in an improved placental morphology, as indicated by a higher placental index and a reduced ratio of the placental labyrinth to the junctional zone, suggesting potential benefits for fetal health. The phenolic compounds in pomegranate juice are known for their antioxidant and anti-inflammatory properties [39]. These compounds can help to reduce oxidative stress and inflammation, which are key contributors to the development and progression of preeclampsia [40]. By mitigating these underlying factors, pomegranate juice can potentially alleviate the symptoms and complications of preeclampsia. These results align with previous studies that have demonstrated the beneficial effects of various natural compounds on L-NAME-induced preeclampsia. For instance, Ilyas et al. showed that kidney necrosis in a preeclampsia rat model was improved by using a turmeric rhizome extract, which is a well-known rich source of curcumin [41], and Ju et al. showed that curcumin ameliorated preeclampsia symptoms by reducing blood pressure, decreasing proteinuria, and improving the placental morphology [9]. Similarly, Caldeira-Dias et al. demonstrated that resveratrol in grape juice had a beneficial effect on endothelial dysfunction in an in vitro preeclampsia model [42]. Lang et al. found that resveratrol led to significant reductions in blood pressure and proteinuria, along with improved placental function and reduced oxidative stress [10].

Moreover, Abdelsalam et al. reported that artemisinin administration resulted in reduced blood pressure, proteinuria, and oxidative stress markers and an improved placental morphology in L-NAME-induced preeclampsia [11]. Artemisinin is a bioactive compound that is extensively found in *Artemisia annua.* It acts as an angiotensin-converting enzyme (ACE) inhibitor and interaction peptide against hypertension [43]. Jain et al. evaluated the antihypertensive activity via ACE inhibition in *Phoenix sylvestris* (L.) Roxb extracts, which are rich in flavonoids and polyphenols, especially quercetin [44]. Yang et al. demonstrated that quercetin supplementation ameliorated preeclampsia symptoms by decreasing blood pressure, proteinuria, and oxidative stress and improving placental function [8]. Lastly, Mazurakova et al. found that plant flavonoids improved blood pressure, reduced proteinuria and oxidative stress, and ameliorated placental dysfunction in a similar model [12].

Studies focusing on kidney function in L-NAME-induced preeclampsia models have shown similarly promising results with natural compounds. For instance, Abdelsalam et al. demonstrated that artemisinin administration not only reduced blood pressure and oxidative stress but also improved renal function, indicating protective effects on the kidneys in preeclampsia [11]. Additionally, Mazurakova et al. found that plant flavonoids improved kidney function parameters and reduced renal oxidative stress markers in L-NAME-induced preeclampsia [12]. Our findings are comparable to these studies, reinforcing the potential of natural compounds in managing preeclampsia. The significant decrease in endocapillary hypercellularity observed in our study, particularly with high-dose pomegranate juice, further confirms the beneficial effects of pomegranate juice at the organ level. These studies suggest that natural compounds, including pomegranate juice as used in our study, may exert protective effects on kidney function in preeclampsia. The antioxidant and anti-inflammatory properties of pomegranate juice’s phenolic compounds are the likely mechanisms underlying these effects, as they can mitigate oxidative stress and inflammation in renal tissue [39,40].

Our findings suggest that pomegranate juice exhibits differential effects on placental and renal recovery, with lower doses being sufficient for placental protection, whereas kidney recovery requires a higher pomegranate juice dose. The lack of a clear dose–response trend in the placenta at high doses may be due to saturation effects, where the maximal therapeutic impact is achieved at moderate pomegranate juice doses, with no further improvement at higher concentrations. This discrepancy between the placental and renal responses suggests that the placenta may be more sensitive to the polyphenolic compounds in pomegranate juice, whereas the kidneys may require prolonged exposure or higher doses to achieve comparable protective effects. These findings support the refinement of the study’s hypothesis, highlighting that, while pomegranate juice effectively mitigates both placental and renal damage, different doses are required for optimal benefits in each organ. This information strengthens the study’s impact by quantitatively demonstrating pomegranate juice’s protective effects and providing a basis for dose optimization in future therapeutic applications.

The findings of this study demonstrate that pomegranate juice effectively mitigates the elevated levels of sFlt-1, a key biomarker of preeclampsia, in a dose-dependent manner. L-NAME treatment significantly increased the sFlt-1 concentrations, consistent with its ability to induce preeclampsia-like symptoms, including placental dysfunction and systemic endothelial stress. Elevated sFlt-1 levels are strongly associated with impaired angiogenesis and vascular dysfunction in preeclampsia, making it a critical target for therapeutic intervention. Pomegranate juice reduced the sFlt-1 levels, with high doses showing the most significant effect, reducing the levels to the near-normal values observed in the control group. This dose-dependent response can be attributed to the high phenolic content of pomegranate juice, particularly punicalagin, the most abundant polyphenol in pomegranate. Punicalagin has been shown to exert potent antioxidant and anti-inflammatory properties, reducing oxidative stress and improving endothelial function, both of which are key contributors to preeclampsia’s pathophysiology. Additionally, punicalagin has been demonstrated to restore the angiogenic balance by upregulating VEGF while downregulating sFlt-1, further supporting its role in alleviating preeclampsia symptoms. Interestingly, the low-dose pomegranate group showed minimal effects on the sFlt-1 levels, highlighting the importance of sufficient bioactive compound concentrations for therapeutic efficacy. Moderate doses demonstrated a partial improvement, particularly in the later stages of gestation, suggesting that prolonged treatment may amplify the benefits of pomegranate juice. High-dose pomegranate juice treatment, however, provided consistent and significant reductions in sFlt-1, underscoring its potential as a natural therapeutic agent. Hypertension and proteinuria in preeclampsia are strongly associated with increased sFlt-1 levels, which impair vascular endothelial function and kidney health. Our findings suggest that pomegranate juice may reduce these symptoms, potentially through modulating the angiogenic balance by lowering the sFlt-1 levels. Punicalagin has also been shown to enhance the placental nitric oxide levels, decrease oxidative stress markers, and improve the placental antioxidant capacity, which may explain the observed improvements in the placental morphology and fetal outcomes in this study.

Taken together, our findings contribute to the growing body of evidence supporting the potential of phenolic-rich pomegranate juice in mitigating the multi-organ manifestations of preeclampsia, including placental and renal dysfunction. The total phenolic content of concentrated pomegranate juice obtained in this study was 5223.97 mg/L, surpassing that reported by Anahita et al. [15]. These heightened phenolic levels are thought to influence underlying pathophysiological mechanisms such as oxidative stress and inflammation, potentially alleviating preeclampsia symptoms and enhancing placental and renal function in our rat model.

## 5. Conclusions

In this study, we evaluated the therapeutic effects of pomegranate juice on preeclampsia, focusing on key parameters such as maternal weight gain, hypertension, proteinuria, placental function, and fetal outcomes. Additionally, we measured the sFlt-1 levels, a key biomarker of preeclampsia, to provide further insights into the mechanisms underlying the beneficial effects of pomegranate juice. Our results demonstrated that pomegranate juice administration significantly mitigated the L-NAME-induced elevation of sFlt-1 levels in a dose-dependent manner, with high-dose pomegranate juice reducing the sFlt-1 concentrations to levels comparable to those in the normal control group. This highlights the role of pomegranate juice in restoring the angiogenic balance and alleviating endothelial dysfunction, both of which are central to preeclampsia’s pathophysiology. The reduction in sFlt-1 levels observed in this study complements the improvements in physiological and morphological outcomes, including reduced hypertension, proteinuria, and improved placental function. These findings provide strong evidence that pomegranate juice’s antioxidant and anti-inflammatory properties, attributed to its phenolic compounds, play a critical role in mitigating oxidative stress and modulating angiogenesis.

Our study adds new insights by demonstrating a dose–response relationship in a controlled experimental model that has not been systematically evaluated in previous studies. Specifically, our findings indicate that different doses of pomegranate juice produce distinct effects in terms of maternal weight regulation, blood pressure, proteinuria, the placental structure, and renal protection. This provides important information for the determination of an optimal therapeutic dose.

Furthermore, our expanded mechanistic analysis suggests that pomegranate juice exerts its beneficial effects through multiple pathways, including the following.

- Nitric Oxide (NO) Pathway: Pomegranate juice has been shown to enhance NO’s bioavailability, promoting vasodilation and improved endothelial function, which are essential in counteracting hypertension and vascular dysfunction in preeclampsia.
- Oxidative Stress Reduction: Polyphenols such as punicalagin and ellagic acid, abundant in pomegranate juice, exhibit strong antioxidant properties, mitigating oxidative stress-induced damage in preeclampsia and reducing endothelial dysfunction.
- Inflammation Modulation: Pomegranate juice possesses anti-inflammatory effects, which may help to reduce inflammatory cytokine levels, thereby influencing the expression of angiogenic factors such as sFlt-1, PlGF, endoglin, and cystatin C.

Given the central role of angiogenic imbalance in preeclampsia, our study focused on sFlt-1 as a key biomarker of endothelial dysfunction. However, accumulating evidence suggests that a broader panel of angiogenic and anti-angiogenic markers, such as PlGF, endoglin, and cystatin C, plays a crucial role in preeclampsia’s pathophysiology and may provide deeper mechanistic insights [45,46]. PlGF is essential for placental vascularization, and a significant decline in its levels is associated with increased disease severity [46]. Similarly, endoglin, a co-receptor of transforming growth factor-beta (TGF-β), has been implicated in endothelial dysfunction and vascular maladaptation in preeclampsia [45]. Additionally, cystatin C, a marker of renal dysfunction, has been linked to impaired placental perfusion and increased oxidative stress in preeclamptic pregnancies [45,46].

While our study focused on the overall therapeutic effects of pomegranate juice, we acknowledge the importance of precise bioactive compound quantification. The Folin–Ciocalteu method was used to assess the total phenolic content (TPC) as an indicator of the antioxidant capacity, which has been widely used in polyphenol-rich natural product research. However, we recognize its limitations in differentiating individual phenolic compounds. Future studies should incorporate advanced chromatographic techniques, such as high-performance liquid chromatography (HPLC) or mass spectrometry, to accurately quantify key active phenolics such as punicalagin, ellagic acid, and anthocyanins [47] and determine their individual contributions to preeclampsia mitigation. These analytical refinements will enhance the mechanistic insights and improve the translational relevance for future clinical applications.

Future studies should explore these mechanisms further by incorporating PlGF, endoglin, and cystatin C alongside other molecular markers, such as VEGF, pro-inflammatory cytokines (TNF-α, IL-6), and oxidative stress indicators (malondialdehyde (MDA), superoxide dismutase (SOD)) [48]. This comprehensive biomarker approach will help to elucidate the precise molecular interactions by which pomegranate juice modulates sFlt-1 expression, restores the angiogenic balance, and improves endothelial function, thereby offering deeper insights into its therapeutic potential for preeclampsia.

Additionally, while this study has successfully demonstrated the effects of pomegranate juice on the sFlt-1 levels, additional analyses are warranted to further characterize the specific active phenolic compounds within pomegranate juice, particularly punicalagin, ellagic acid, and anthocyanins, and their precise contributions to preeclampsia mitigation. Future research should also determine the optimal dosing regimen and investigate the long-term safety and efficacy of pomegranate juice in clinical settings. Expanding these findings to human studies would provide valuable insights into pomegranate juice as a natural therapeutic intervention for preeclampsia.

## Figures and Tables

**Table 1:** The calculation of the pomegranate juice dosage for the experimental rats.

Step	Final Value	Final Value
Human Dose (TPC-based) [23]	1685 mg/L × 500 mL ÷ 60 kg	28.08 mg/kg/day
Animal Volume Dose [23]	500 mL ÷ 60 kg	8.33 mL/kg/day
Animal Dose (TPC-based) [23]	28.08 mg/kg ÷ 2 mg/mL	14.04 mL/kg/day
HED Calculation [25]	14.04 × (37/6)	86.57 mg TPC/kg
Adjusted HED [26]	86.57 ÷ 1.53	56.58 mg TPC/kg
Final Rat Dosage	(56.58 mg × 1000 mL) ÷ 5223.97 mg	11 mL/kg/day
OECD Maximum Dosage [24]	56.58 ÷ 1.53	36.98 mg/kg (High Dose Group)

**Table 2:** Pomegranate juice dosages for experimental animals.

Dosage Category	HED (mg TPC/L)	Concentrated Pomegranate Juice (mL)	Distilled Water (mL)
Maximum dosage for experimental animals from Ruamthum [26]	56.58	11.0	0.0
Adjusted according to OECD Guidelines			
High-dose pomegranate juice	36.98	7.2	3.8
Medium-dose pomegranate juice	24.17	4.7	6.3
Low-dose pomegranate juice	15.80	3.1	7.9

HED = Human Equivalent Dose, TPC = total phenolic content, OECD = Organisation for Economic Cooperation and Development.

## Data Availability

The original contributions presented in the study are included in the article, and further inquiries can be directed to the corresponding authors. The data are not publicly available due to institutional policy on data confidentiality and ethical considerations involving animal research.

## Supplementary Materials

The following supporting information can be downloaded at: https://www.mdpi.com/article/10.3390/nu17071143/s1, Supplementary Material: Detailed Step-by-Step Dosage Calculation.
