# Supplementary material for: Pomegranate Juice Alleviates Preeclampsia Symptoms in an L-NAME-Induced Rat Model: A Dose-Dependent Study
Source: Nutrients. 2025 Mar 26;17(7):1143. doi: 10.3390/nu17071143 (PMC11990272; doi:10.3390/nu17071143)
Supplement: Supplementary file 1 [file nutrients-17-01143-s001.zip › nutrients-3499743-supplementary.pdf]

Supplementary Material: Detailed Step-by-Step Dosage Calculation

Calculation of pomegranate juice dosage for experimental animals

The dosage of pomegranate juice for experimental animals in this study was calculated based on the total phenolic content (TPC) in pomegranate juice, following the approach used in a previous study by Dujaili et al. [23]. The calculations were adjusted using the Human Equivalent Dose (HED) method based on body surface area, as recommended by the Center for Drug Evaluation and Research (CDER) [25], and further refined according to the OECD Guideline for Testing of Chemicals Test No. 415 [24].

Step 1: Determination of human dose based on total phenolic content (TPC)

From the Dujaili et al. [23] study, participants received:

- 500 mL of pomegranate juice per day
- Total phenolic content (TPC): 1685 mg/L

Convert Human Dose to mg/kg for a 60 kg Person:

Animal Dose (mg/kg) = [Total Phenolic Content (mg/L) × Volume (L)] ÷ [Human Weight (kg)]

= [1685 (mg/L) x 0.5 (L)] ÷ [60 (kg)]

= 842.5 ÷ 60

= 28.08 mg/kg/day

Thus, the estimated total phenolic intake for a human is 28.08 mg/kg/day.

Step 2: Convert human dose to animal dose using volume for rats

According to Dujaili et al. [23], 500 mL/day for a 60 kg human corresponds to:

500 (mL/day) ÷ 60 (kg) = 8.33 mL/kg/day

However, using the total phenolic content approach:

Animal Dose (mL/kg/day) = [Human Dose (mg/kg/day)] ÷ [TPC in Juice (mg/mL)]

= 28.08 ÷ 2

= 14.04 mL/kg/day

This 14.04 mL/kg/day value was then used to calculate the Human Equivalent Dose (HED).

Step 3: Convert animal dose to human equivalent dose (HED)

Using the CDER formula for dose conversion between species [25]:

HED (mg/kg) = [Animal dose (mg/kg)] × [Animal Km ÷ Human Km]

where:

- Animal Km (rat) = 6
- Human Km = 37

HED = 14.04 × (37 ÷ 6)

= 14.04 × 6.167

= 86.57 mg TPC/kg

Thus, the estimated HED for a 60 kg human is 86.57 mg TPC/kg.

Step 4: Adjusting for toxicity study findings

The HED calculated from Dujaili et al. [23] was further adjusted based on an acute toxicity study by Ruamthum [26], applying a safety factor of 1.53:

Adjusted HED = 86.57 ÷ 1.53

= 56.58 mg TPC/kg

Using this adjusted value, the appropriate dosage of pomegranate juice for experimental animals was set at 13 mL/kg/day.

Step 5: Final dosage calculation for this study

In this study, the total phenolic content (TPC) measured in the pomegranate juice was 5223.97 mg GAE/L. To achieve the required 56.58 mg of total phenolics, the appropriate rat dosage was calculated as follows:

Dosage (mL/kg) = (56.58 × 1000) ÷ 5223.97

= 56580 ÷ 5223.97

= 11 mL/kg/day

Thus, the final dosage administered to rats in this study was 11 mL/kg/day.

Step 6: Maximum dosage based on OECD Guidelines

According to the OECD Guideline for Testing of Chemicals Test No. 415 [24], the maximum oral dosage for pregnant animals should not exceed 1000 mg/kg. To comply with this safety limit, the maximum dosage for pregnant rats in this study was further adjusted:

Maximum Dose = 56.58 ÷ 1.53

= 36.98 mg TPC/kg

Thus, the high-dose group received 36.98 mg/kg, with dosages proportionally reduced for the medium- and low-dose groups.
